# Ratio of Creatine Kinase to Alanine Aminotransferase as a Biomarker of Acute Liver Injury in Dystrophinopathy

**DOI:** 10.1155/2018/6484610

**Published:** 2018-06-19

**Authors:** Liang Wang, Menglong Chen, Min Xu, Jing Li, Pinning Feng, Ruojie He, Yuling Zhu, Huan Li, Jinfu Lin, Cheng Zhang

**Affiliations:** ^1^Department of Neurology, National Key Clinical Department and Key Discipline of Neurology, The First Affiliated Hospital, Sun Yat-sen University, Guangzhou, Guangdong, China; ^2^Department of Neurology, Guangzhou Overseas Chinese Hospital, Guangzhou, Guangdong, China; ^3^Department of Dermatology, Sun Yat-sen Memorial Hospital, Sun Yat-sen University, Guangzhou, Guangdong, China; ^4^Department of Clinical Laboratory, The First Affiliated Hospital, Sun Yat-sen University, Guangzhou, Guangdong, China

## Abstract

**Objective:**

To investigate the ratios of creatine kinase (CK) to aminotransferases as biomarkers of acute liver injury in dystrophinopathy.

**Methods:**

C57 and mdx (dystrophic) mice were treated with a hepatotoxic reagent D-galactosamine (D-GalN). The degrees of liver and muscle injury were assessed using histological examinations. To examine whether serum CK-adjusted aminotransferase levels could indicate liver status in dystrophic mice, the CK/alanine aminotransferase (ALT) and CK/aspartate aminotransferase (AST) ratios were analyzed. Furthermore, we enrolled 658 male patients with dystrophinopathy and 378 male patients without muscle and liver injury as control, whose serum ALT, AST, and CK levels were examined.

**Results:**

Animal experiments indicated that D-GalN treatment could induce acute liver injury but not muscle injury. Additionally, D-GalN decreased the CK/ALT and CK/AST ratios in both C57 mice and mdx mice (*P* < 0.001). However, there was an overlap of the CK/AST ratio between dystrophic mice with and without acute liver injury. In patients with dystrophinopathy, CK-adjusted ALT diminished the variability associated with age, genotype, clinical phenotype, and motor function (*P* > 0.05).

**Conclusions:**

CK/ALT is a potential biomarker for the differential evaluation of acute liver injury in dystrophic mice, which highlights the value to further evaluate the practice of CK/ALT in dystrophinopathy patients.

## 1. Introduction

Dystrophinopathy is the most common type of muscular dystrophy characterized by progressive necrosis, fibrosis, fatty tissue replacement, inflammation, and loss of regenerative capacity in skeletal and cardiac muscles [1, 2]. According to the disease course, it can be divided into Duchenne muscular dystrophy (DMD), intermediate muscular dystrophy (IMD), and Becker muscular dystrophy (BMD) [3]. Eighty percent of patients have large genetic rearrangements in the *DMD* gene encoding dystrophin, and the rest suffer from small mutations [4]. Glucocorticoid administration can ameliorate the progression of the disease but cannot cure it [5]. Although dystrophinopathy still has no cure and most patients die of heart and respiratory failure, effective health measures that improve life quality and expectancy in patients with dystrophinopathy have been developed in the last 20 years [5].

Health management has focused on the functions of critical organs such as the heart, lung, and kidney, and specific biomarkers to monitor their functional activity have been suggested [6–8]. However, less attention was paid to the liver, which can also be injured in dystrophinopathy. Acute liver failure has been reported in a dystrophinopathy patient treated with paracetamol [9]. Liver failure can also be a significant postoperative complication after tendon lengthening, scoliosis surgery, and heart transplantation which are performed in patients with dystrophinopathy [10–15]. In addition, as an increasing number of preclinical studies and clinical trials for promising treatments such as gene therapy and stem cell therapy have been conducted, it is important to evaluate hepatotoxicity as a criterion of therapeutic safety [16, 17]. Therefore, it is necessary to evaluate liver conditions in dystrophinopathy using reliable diagnostic methods.

Aminotransferases, including alanine aminotransferase (ALT) and aspartate aminotransferase (AST), are widely used in evaluating liver status because of high diagnostic accuracy and low test costs [18]. However, although aminotransferase activity reflects hepatocyte injury [19], there are limitations for its application in dystrophinopathy, such as elevated background levels and age-dependent variability caused by the release of aminotransferases from injured myofibers [20]. Previous studies indicate that serum gamma-glutamyl transferase levels can be a valuable diagnostic tool for screening liver disease in dystrophinopathy; however, its sensitivity is limited, especially in patients with noncholestatic, hepatocellular conditions [21, 22]. Although imaging modalities such as sonography represent well-developed methods to estimate liver status, they are time- and cost-consuming and not appropriate for screening. Therefore, considering an effective worldwide application of serum aminotransferase activity to assess liver injury, aminotransferase levels adjusted by some indexes specifically reflecting muscle injury could be used to evaluate liver status in dystrophinopathy.

Compared to aminotransferases, creatine kinase (CK) is a more specific index of muscle injury [20]; therefore, we hypothesized that CK-adjusted aminotransferase levels may present an even more accurate biomarker of liver damage in dystrophinopathy. In this study, we aimed to establish a mouse model of dystrophinopathy with acute liver injury and examined whether the CK-adjusted aminotransferase levels can indicate acute liver injury in this model. Additionally, a preliminary investigation for the applicability of CK-adjusted aminotransferases in patients with dystrophinopathy was undertaken.

## 2. Methods

### 2.1. Establishment of Animal Models for Acute Liver Injury

All experiments were approved by the ethics committee for Clinical Research and Trials of the First Affiliated Hospital of Sun Yat-sen University. C57BL (C57) and C57BL/10ScSn-Dmdmdx/J (mdx, a mouse model of dystrophinopathy) male mice aged 4-5 weeks were obtained from the Guangdong Medical Laboratory Animal Center (Guangzhou, GD, China) and the Model Animal Research Center of Nanjing University (Nanjing, JS, China), respectively. D-Galactosamine (D-GalN), an amino sugar resulting in depletion of uridine moieties within the liver and leading to hepatocyte necrosis, was chosen as an inducer of acute liver injury [23, 24]. Mice were randomly divided into four groups: C57 NS, C57 D-GalN, mdx NS, and mdx D-GalN (*n* = 10 each). NS and D-GalN mice were injected intraperitoneally with normal saline (NS; China Otsuka Pharmaceutical Company, Tianjin, China) or 2000 mg/kg D-GalN (Sigma-Aldrich, St. Louis, MO, USA) dissolved in NS for inducing hepatotoxicity, respectively [25, 26]. The total injection volume per mouse was 20 mL/kg. After 24 h, blood samples were obtained via tail cut; then, mice were sacrificed by cervical vertebrae dislocation and their livers and tibialis anterior (TA) muscles were extracted.

### 2.2. Evaluation of Muscle and Liver Lesions

TA is one of the most common muscles used to evaluate muscle lesions in mdx mice as it is easy to extract; therefore, it was used to assess myotoxicity of D-GalN [27, 28]. TA muscles were treated and sectioned and stained with hematoxylin-eosin (HE), adenosine triphosphatase (ATPase) following preincubation pH at 10.6, and nicotinamide adenine dinucleotide tetrazolium reductase (NADH-TR) for morphological analysis as previously described [27, 29]. Myofiber types were classified into type I myofibers (light staining) and type II myofibers (dark staining) using ATPase (pH 10.6) staining. Additionally, myofibers stained with NADH-TR were classified into type I myofibers (dark staining), type IIA myofibers (moderate staining), and type IIB myofibers (light staining). Overall, fiber types were determined with a combination of ATPase staining and NADH-TR staining. Necrotic myofibers, regenerating myofibers, centronucleated myofibers, and a ratio of central versus peripheral nuclei were counted to evaluate necrosis and degeneration/regeneration cycles of TA [30].

Mouse livers were fixed in neutral-buffered formalin, embedded in paraffin, sectioned, and stained with HE as previously described [31]. The histological severity of liver lesions was graded according to the Ishak system using the sum of necroinflammatory scores A, B, C, and D [32].

### 2.3. Study Participants

Overall, 658 male patients with dystrophinopathy [6 years (4–9)] followed up in our hospital from 2008 to 2016 were included in the study. And 378 male patients without muscle and liver injury [10 years (5–14.75)] were also included as a control. Approval to waive informed parental consent was granted by the ethics committee of the First Affiliated Hospital, Sun Yat-sen University. The study protocol conformed to the ethical guidelines of the 1975 Declaration of Helsinki. The patients did not exhibit outward clinical manifestations of liver disease, and those with abnormal serum total bilirubin were not included. All dystrophinopathy patients were diagnosed with clinical manifestations, biochemical changes, and molecular/histological diagnosis. Twenty patients were given histological diagnosis (antidystrophin immunohistochemistry staining in muscle biopsy samples), which showed absence or low levels of dystrophin. And the others (*n* = 638) were given genetic diagnosis. Serum levels of CK, ALT, and AST were examined; typically, the earliest recorded test results were analyzed. Detailed information is shown in Table 1.

### 2.4. Genotyping

*DMD* genotyping was performed for 638 patients. Multiplex ligation-dependent probe amplification (MLPA) (*n* = 553) and hotspot region polymerase chain reaction (*n* = 11) were used to detect large genetic rearrangements (deletions or duplications), and next-generation sequencing was used to detect small mutations.

### 2.5. Determination of Clinical Phenotypes

Clinical phenotypes were classified according to the age at which walking ability was lost as previously described [3]: DMD, losing walking ability by the age of 13 years; IMD, losing walking ability between 13 and 16 years; and BMD, walking after 16 years. Patients younger than 16 years who retained walking ability were evaluated according to disease severity, serum indexes, muscle biopsy, and molecular tests. Patients who were too young for evaluation were followed up and evaluated later. Patients whose phenotype could not be determined were considered having IMD in this study.

### 2.6. Determination of Serum Enzyme Activity

Approximately 200 *μ*L of blood from each mouse or 5 mL of blood from each patient was collected, centrifuged at 1810 ×g for 5 min at 24°C, and analyzed for ALT, AST, and CK activities using a Beckman Coulter AU5800 clinical chemistry analyzer (ALT: lactate dehydrogenase method, AST: malate dehydrogenase method, and CK: enzymatic method; Beckman Coulter, Brea, CA, USA). To determine CK levels, serum samples were diluted step-wise 3-, 5-, 10-, and 20-fold because CK activity was beyond the linear range, and the CK level was measured and recorded at the highest dilution.

### 2.7. Statistical Analysis

Statistical analysis was performed using SPSS version 20.0 (IBM Corp., Chicago, IL, USA) and GraphPad PRISM version 7.01 (GraphPad Software, San Diego, CA, USA). Normal distribution of variables was tested by the Shapiro-Wilk test (*n* ≤ 50) or Kolmogorov-Smirnov test (*n* > 50); variables with normal distributions and without normal distributions were presented as the mean and standard deviation (median ± standard deviation) and the median and interquartile range [median (interquartile range)], respectively. To analyze differences between two variables and more than three variables with normal distributions, Student's *t*-test or one-way analysis of variance was used. To analyze differences between two variables and more than three variables with nonnormal distributions, the Mann-Whitney *U* test or Kruskal-Wallis *H* test was used. Differences among more than three variables were analyzed using the Bonferroni method. The correlations between two variables with normal distributions and without normal distributions were analyzed by the Pearson method and Spearman method, respectively. All tests were two-tailed, and *P* < 0.1 was considered statistically significant in the Shapiro-Wilk and Kolmogorov-Smirnov tests, whereas *P* < 0.05 was considered significant in other tests.

## 3. Results

### 3.1. Muscle Necrosis and Regeneration in Mice

**Figure 1:**
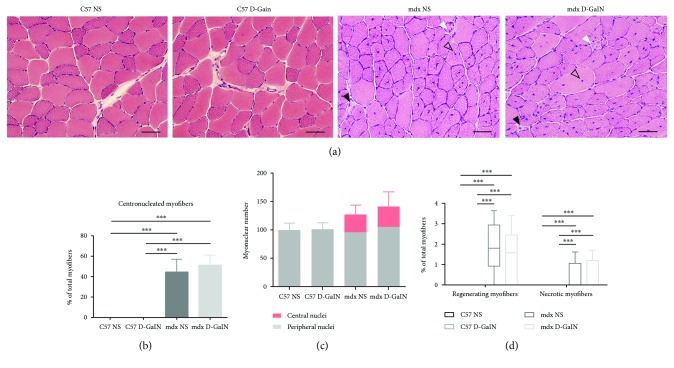
Necrosis and regeneration in the muscle of D-galactosamine-treated and D-galactosamine-untreated mice. Mice were intraperitoneally injected with D-galactosamine (D-GalN) or normal saline (NS), and tibialis anterior (TA) was sectioned for histological analysis 24 h later. (a) HE staining showed normal morphology of TA in both the C57 NS and the C57 D-GalN groups and muscle lesions in both the mdx NS and the mdx D-GalN groups. Solid black arrows: necrotic myofibers with pale and homogenous sarcoplasm and pyknotic nuclei; solid white arrows: regenerating myofibers with a small diameter and vesicular central nuclei; and hollow black arrows: centronucleated myofibers. Scale bar: 100 *μ*m. (b, c) The percentage of centronucleated myofibers in total myofibers (b) and the ratio of central versus peripheral nuclei (c) of different groups. (d) The percentage of regenerating and necrotic myofibers in total myofibers of different groups. ^∗∗∗^*P* < 0.001.

No mice died after 24 h of D-GalN treatment. To detect the myofiber necrosis and regeneration in mice, TA sections were stained with HE. The results showed that necrosis, regeneration, and centronucleated myofibers were observed in mdx mice but not in C57 mice (Figure 1(a)). Additionally, muscle lesions were evaluated using the percentage of centronucleated myofibers, the ratio of central versus peripheral nuclei, and the percentage of regenerating or necrotic myofibers [30]. The differences of above indexes were significant between C57 NS mice and mdx NS mice, and the same was true for C57 D-GalN mice and mdx D-GalN mice (*P* < 0.001, Figures 1(b)–1(d)). However, these differences could not be observed between the D-GalN treated group and the untreated group in C57 mice or mdx mice (*P* > 0.05).

### 3.2. Myofiber Types in Mice

**Figure 2:**
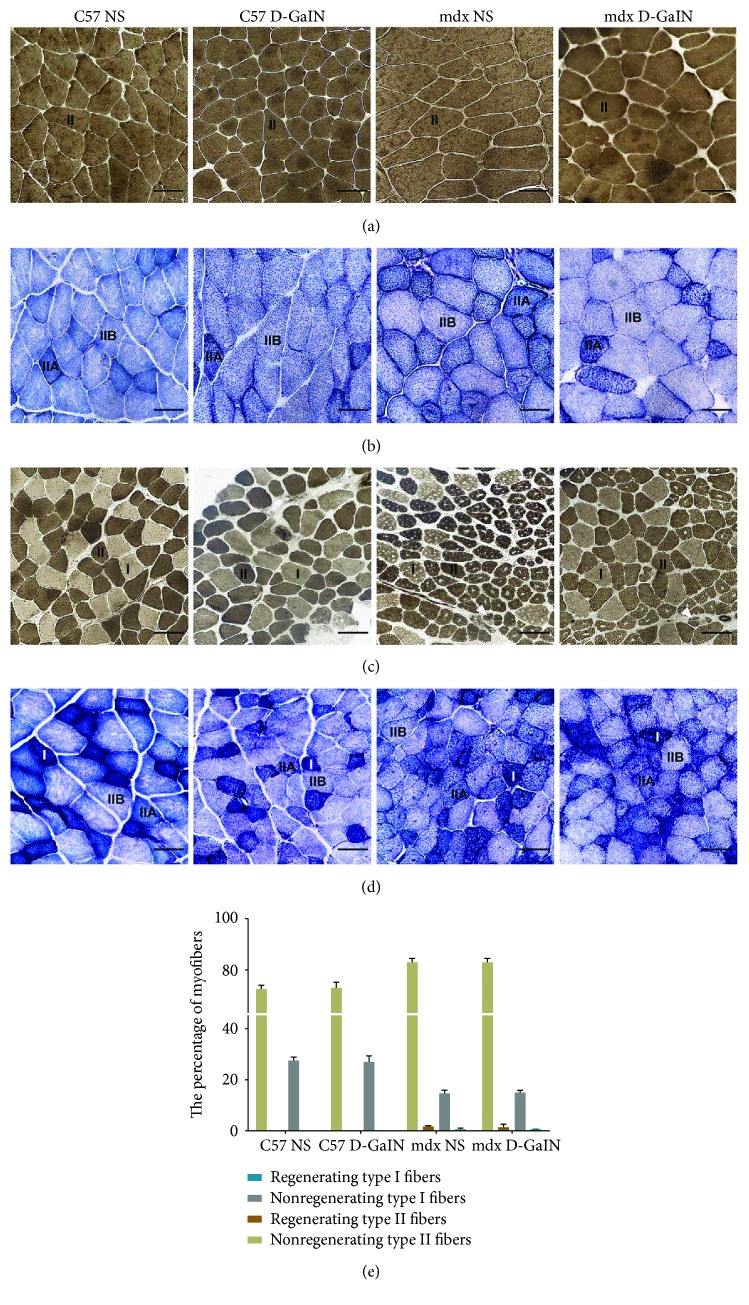
Myofiber types in the muscle of D-galactosamine-treated and D-galactosamine-untreated mice. (a, b) Superficial area of tibialis anterior (TA). (c, d) Deep area of TA. (a, c) Adenosine triphosphatase activity following preincubation pH at 10.6; solid white arrows: regenerating myofibers. (b, d) Nicotinamide adenine dinucleotide tetrazolium reductase. Scale bar: 50 *μ*m. (e) The percentage of different myofiber types in four mouse groups.

Myofibers could be mainly classified into two groups: type I myofibers and type II myofibers, of which type II consisted of type IIA and type IIB myofibers. The changes of the proportion of fiber types indicated muscle injury. To detect the fiber type changes and regeneration of different myofiber types in mice, TA sections were stained with ATPase and NADH-TR. The results indicated that almost all myofibers in the superficial area of TA were type II myofibers in both C57 and mdx mice with or without D-GalN treatment (Figures 2(a), 2(b)). Additionally, type II myofibers were also dominant in the deep area of TA, whose proportion was higher in mdx mice than in C57 mice, but D-GalN treatment did not cause an extra increase of type II myofibers neither in C57 nor in mdx mice (72.46% ± 1.43% in C57 NS versus 72.93% ± 2.23% in C57 D-GalN versus 84.66% ± 1.46% in mdx NS versus 84.25% ± 0.83% in mdx D-GalN, *P* < 0.001, Figures 2(c)–2(e)). Then, we analyzed the regeneration of different fiber types in the deep area of TA in mdx mice. We found that regeneration toward type II myofibers was more common than that toward type I myofibers (*P* < 0.001, Figure 2(e)), which could be observed in both D-GalN-treated and D-GalN-untreated mdx mice.

### 3.3. D-GalN-Induced Hepatic Necrosis in Mice

**Figure 3:**
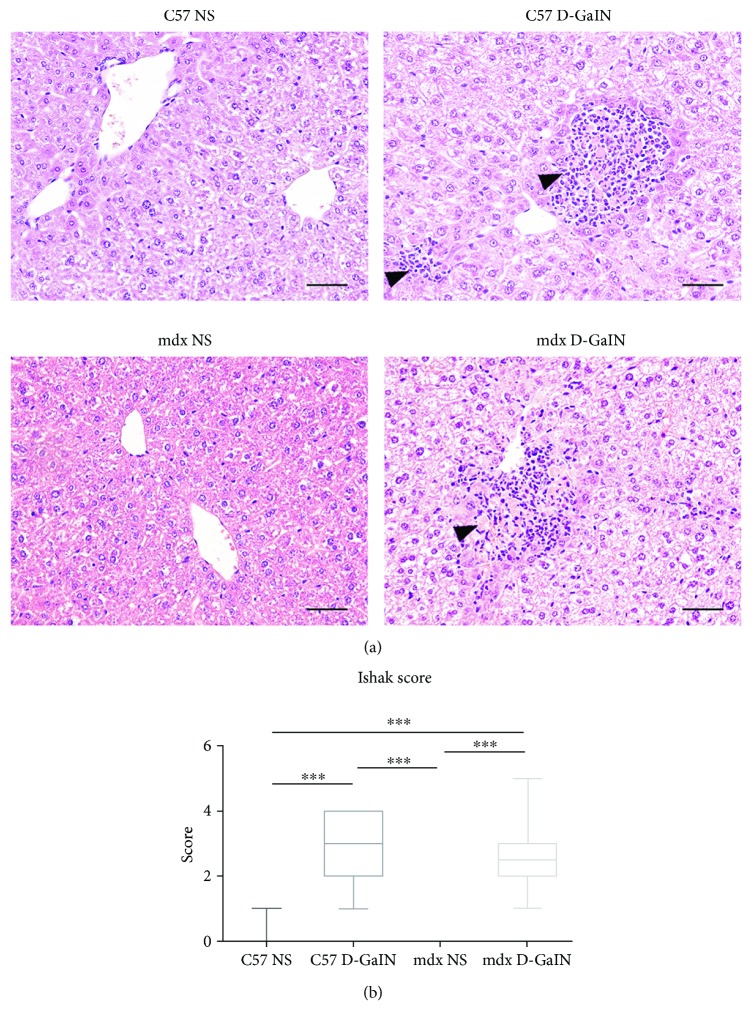
Liver necrosis in D-galactosamine-treated and D-galactosamine-untreated mice. (a) HE staining revealed necrotic lesions (hepatic necrosis and inflammation) in the liver of D-GalN-treated mice (solid black arrows). Scale bar: 100 *μ*m. (b) Ishak (pathological) scores of liver lesions. ^∗∗∗^*P* < 0.001.

Liver histology was evaluated to determine liver injury. The results indicated that obvious hepatocyte necrosis could be observed in D-GalN-treated mice (Figure 3(a)). A normal hepatic lobule structure consisted of a regular arrangement of hepatic cells radially around the central vein. The hepatocytes appeared to be necrotic, and inflammation in lesions was observed in 2000 mg/kg D-GalN-treated C57 mice and mdx mice (*P* < 0.001, Figure 3(b)). Overall, above results indicated that D-GalN caused acute liver injury but no muscle injury in mdx mice; thus, the mouse model of dystrophinopathy with acute liver injury was established successfully.

### 3.4. The Ratio of CK to Aminotransferases Decreased in Mice with Acute Liver Injury

**Figure 4:**
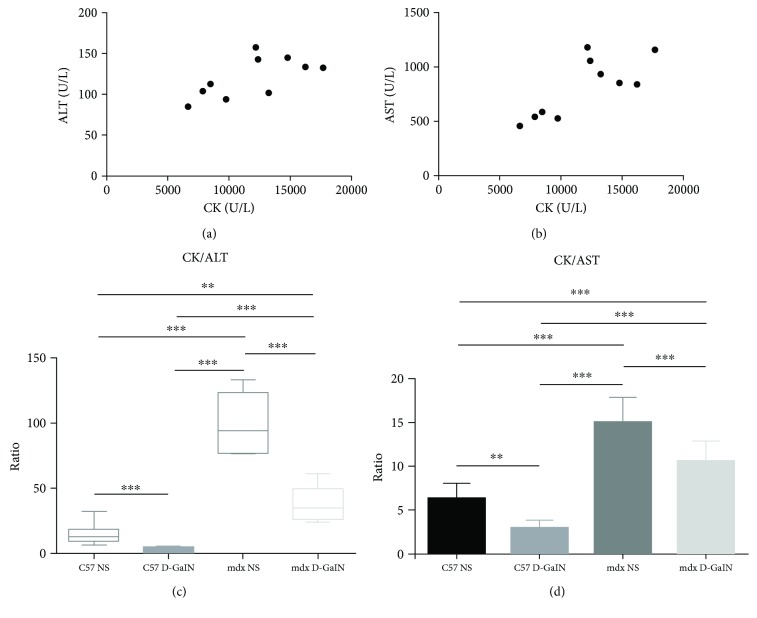
The ratio of creatine kinase to aminotransferase in D-galactosamine-treated and D-galactosamine-untreated mice. (a, b) The scatter plots of creatine kinase (CK) versus alanine aminotransferase (ALT (a)) and CK versus aspartate aminotransferase (AST (b)) in D-GalN-untreated mdx mice. (c, d) The ratio of CK to ALT (c) and CK to AST (d) in four mouse groups. ^∗∗^*P* < 0.01 and ^∗∗∗^*P* < 0.001.

To verify whether CK-adjusted aminotransferases could detect acute liver injury, serum CK, ALT, and AST were analyzed in mice. In the mdx control group (mdx NS), the linear correlation between levels of CK and ALT was observed (*P* < 0.05, *r* = 0.65, Figure 4(a)). Similarly, AST level was also correlated with CK level (*P* < 0.01, *r* = 0.77, Figure 4(b)). Thus, the ratios of CK to aminotransferases were calculated as CK-adjusted aminotransferases. A significant decrease in CK/ALT and CK/AST could be observed in D-GalN-treated mice compared to control (*P* < 0.001, Figures 4(c) and 4(d)). Furthermore, a significant difference in CK/ALT was observed among the four mouse groups: 12.50 (9.33–18.33) in C57 NS versus 2.50 (1.68–4.43) in C57 D-GalN versus 94.40 (76.73–123.85) in mdx NS versus 34.70 (25.98–49.78) in mdx D-GalN (*P* < 0.001, Figure 4(c)). Similarly, a significant difference in CK/AST was also observed among the four mouse groups: 6.33 ± 1.74 in C57 NS versus 2.95 ± 0.98 in C57 D-GalN versus 15.04 ± 2.83 in mdx NS versus 10.65 ± 2.19 in mdx D-GalN (*P* < 0.001, Figure 4(d)). However, a partial overlap of CK/AST could be observed between mdx mice with and without acute liver injury. In conclusion, these results indicated that the CK/ALT and CK/AST could be used to distinguish among normal liver status, acute injury, and dystrophinopathy without and with acute liver injury. Additionally, CK/ALT was more reliable because there was no overlap of CK/ALT between mice with and without acute liver injury.

### 3.5. ALT Adjustment by CK Can Diminish Age Variability in Patients with Dystrophinopathy

**Figure 5:**
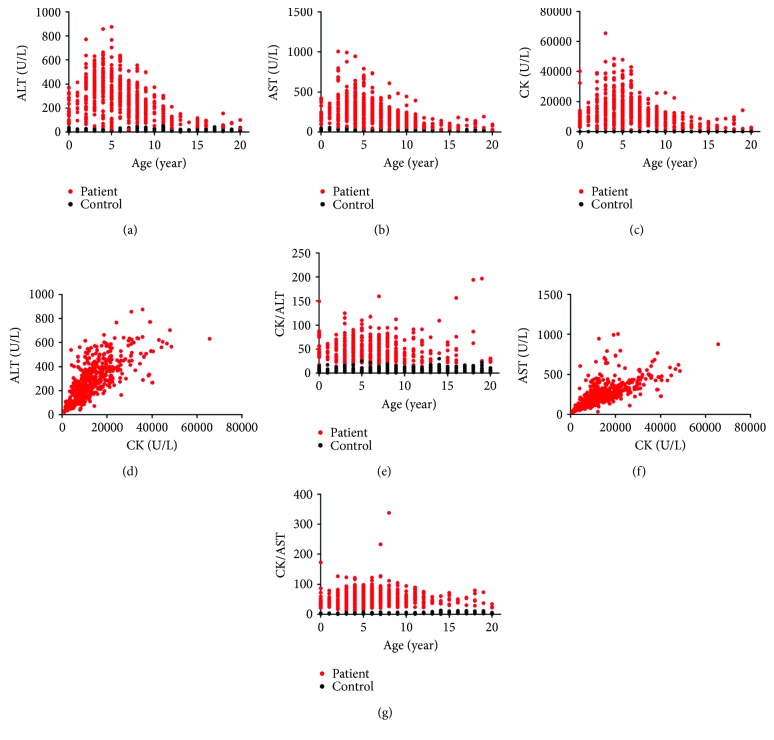
Scatter plots of enzyme activity profiles in patients with dystrophinopathy. (a–c) Age versus alanine aminotransferase (ALT (a)), aspartate aminotransferase (AST (b)), and creatine kinase (CK (c)). (d) CK versus ALT. (e) Age versus CK/ALT. (f) CK versus AST. (g) Age versus CK/AST.

To investigate the possible applicability of CK/ALT for patients with dystrophinopathy, we analyzed enzyme profiles in patients of different ages. ALT, AST, and CK levels in dystrophinopathy patients peaked at the age of 5 years (Figures 5(a)–5(c)). Considering that CK could specifically reflect muscle injury, the levels of aminotransaminases were adjusted to those of CK [20]. The linear correlation between ALT and CK was significant (*P* < 0.001, *r* = 0.75, Figure 5(d)); therefore, we used the CK/ALT ratio to diminish age-dependent fluctuations and found that the distribution of CK/ALT did not differ between patients of different ages (*P* = 0.56, Figure 5(e)). A similar linear correlation was also observed between AST and CK (*P* < 0.001, *r* = 0.79, Figure 5(f)), but the age-dependent difference in CK/AST was significant (*P* < 0.001, Figure 5(g)). Thus, our results indicated that the CK/ALT could diminish age variability in dystrophinopathy. Furthermore, CK/ALT could also diminish variability associated with genotype, clinical phenotype, and motor function (data not showed). The 5th percentile for CK/ALT was determined by SPSS software as 22.16.

## 4. Discussion

In this study, we established a mouse model of dystrophinopathy with acute liver injury. The results showed that CK/ALT and CK/AST ratios decreased significantly in this model and the overlap of CK/AST ratios between dystrophic mice with and without acute liver injury resulted in the possibility of false discrimination. In addition, the CK/ALT ratio was independent of age and factors associated with muscle injury in patients with dystrophinopathy.

Serum-based enzyme tests have been widely used for various applications in the clinic [33–35]. And a variety of serum enzymes, such as CK, ALT, and AST, were reported to increase in muscular dystrophy [36, 37]. The elevation of serum enzymes in dystrophinopathy is thought to be due to their release from injured myofibers rather than to liver injury as indicated by liver biopsy in patients [38]. It is consistent with the absence of liver damage in D-GalN-untreated mdx mice observed in this study. The elevation of CK and aminotransferase levels in dystrophinopathy is easy to understand due to the organ distribution of enzymes: ALT and AST are distributed mostly in the liver and muscles, while CK is distributed highly in the muscles [18–20, 39–41].

Although aminotransferases are widely applied in the clinic as sensitive biomarkers for hepatocyte injury, the concurrence of increased serum CK and aminotransferases is indicative for muscle injury in most cases in dystrophinopathy [20, 42, 43]. However, the elevated levels of aminotransferases in muscular dystrophy sometimes make clinicians confused, which leads to the misdiagnosis of liver diseases and unnecessary liver biopsy [20, 38]. Thus, the lack of specificity limits the application of aminotransferases in muscular dystrophy. Up till now, hepatocyte injury is still inconvenient to detect in dystrophinopathy due to the lack of excellent highly tissue-specific indexes [19, 21, 22], which results in the challenges of an accurate differential diagnosis of liver injury in dystrophinopathy. Recently, a combination of several serum enzymes to distinguish five types of muscular dystrophy suggested the value of comprehensive evaluation of serum enzyme-based tests for diagnostic purpose in muscular dystrophy [44]. The study gives us a hint that the combination of muscle biomarker and aminotransferases together may be more applicable for the evaluation of hepatocyte injury in dystrophinopathy.

CK is the well-known biomarker closely associated with muscle injury and muscular dystrophy, and elevated levels of CK can differentiate some types of muscular dystrophy [43]. Thus, we chose CK to adjust the baselines of aminotransferases in this study. Considering the obvious linear correlations between CK and aminotransferases in both dystrophic mice and patients with dystrophinopathy, the ratio of CK to aminotransferases may be an appropriate adjustment. Our results indicated that the CK/ALT ratio decreased significantly in dystrophic mice with acute liver injury; thus, decreased CK/ALT could indicate acute liver injury in mice.

In patients with dystrophinopathy, the elevated levels of CK and aminotransferases can vary with age and other factors, such as genotype, clinical phenotype, and motor function, all of which made it difficult to establish applicable baselines of CK and aminotransferase levels for diagnostic purposes [43]. Interestingly, we found that CK-adjusted ALT could diminish the variability associated with age and other factors correlated with muscle injury, which suggests that CK/ALT is less influenced by muscle injury and has the possibility of application for dystrophinopathy. Even though, we need to notice that levels of CK and aminotransferases may be affected by other factors, such as gender, ethnicity, life style, activity levels, surgical procedures, seasonal influences, and medication administration [43, 45], which can lead to an inaccurate diagnosis of acute liver injury. These factors can be easily controlled in a mouse model, but the influence of them must be evaluated when applied in human.

Although AST also had a linear correlation with CK, CK/AST had an overlap between dystrophic mice with and without acute liver injury. And furthermore, CK/AST was still affected by age and factors associated with muscle injury severity in patients, as evidenced by its increase in patients with more severe genotype and phenotype. Compared to ALT, AST has higher concentration in muscle tissue; consistently, it was observed that the increase of serum AST was higher than that of serum ALT after muscle exercise stress [46, 47]. Therefore, serum AST is a less specific indicator of liver injury [42]. Overall, these data suggest that CK/ALT is better for the differential evaluation of acute liver injury in dystrophinopathy.

Mdx mouse is a widely used mouse model of dystrophinopathy, which contributes to numerous understanding of dystrophinopathy [48]. However, a mouse is not a man. The scale, growth pattern, and cell biology of mouse are highly different from human [49, 50]. Besides, as we observed in this study, the distribution of myofiber types in mice is largely different from that in human. Not only limited to TA, higher frequencies of type II myofibers are observed in numerous muscles of mouse compared with human, which adapts to the motor pattern of mouse [50–52]. Additionally, the phenotype and lifespan shortening of mdx mice are milder compared with those of patients [53]. Although the TA of mdx mice exhibits similar pathological changes of dystrophinopathy, such as necrosis, regeneration, various myofiber sizes, and centronucleated myofibers, they are milder and lack obvious fibrosis compared to pathological changes of skeletal muscles in patients [53]. Therefore, we must be cautious to extend the conclusion from animal to human. In this study, the utility of CK/ALT for detecting acute liver injury was verified in dystrophic mice. Although we preliminarily investigated the possibility of CK/ALT for clinical application and determined the lower limit of normal (LLN) of CK/ALT in patients with dystrophinopathy, a further study based on patients' data is necessary before its application in the clinic.

In conclusion, we successfully established a mouse model of dystrophinopathy with acute liver injury. And CK/ALT is a potential biomarker for the differential evaluation of acute liver injury in dystrophic mice, which highlights the value to further evaluate the practice of CK/ALT in patients with dystrophinopathy.

## Figures and Tables

**Table 1:** Clinical information of patients with dystrophinopathy.

Parameters	Number	Percentage
Ethnicity	658	
East Asian	658	100
Clinical phenotype	658	
DMD	464	70.52
IMD	37	5.62
BMD	157	23.86
Muscle biopsy and dystrophin staining	20	
Mutation analysis	638	
Deletion	468	73.35
Duplication	96	15.05
Small mutations	74	11.60

DMD: Duchenne muscular dystrophy; IMD: intermediate muscular dystrophy; BMD: Becker muscular dystrophy.
